# Staffing level: a determinant of late-onset ventilator-associated pneumonia

**DOI:** 10.1186/cc5974

**Published:** 2007-07-19

**Authors:** Stéphane Hugonnet, Ilker Uçkay, Didier Pittet

**Affiliations:** 1 Infection Control Program, University of Geneva Hospitals, Rue Micheli-du-Crest, 1211 Geneva 14, Switzerland

## Abstract

**Introduction:**

The clinical and economic burden of ventilator-associated pneumonia (VAP) is uncontested. We conducted the present study to determine whether low nurse-to-patient ratio increases the risk for VAP and whether this effect is similar for early-onset and late-onset VAP.

**Methods:**

This prospective, observational, single-centre cohort study was conducted in the medical intensive care unit (ICU) of the University of Geneva Hospitals. All patients who were at risk for ICU-acquired infection admitted from January 1999 to December 2002 were followed from admission to discharge. Collected variables included patient characteristics, admission diagnosis, Acute Physiology and Chronic Health Evaluation II score, co-morbidities, exposure to invasive devices, daily number of patients and nurses on duty, nurse training level and all-site ICU-acquired infections. VAP was diagnosed using standard definitions.

**Results:**

Among 2,470 patients followed during their ICU stay, 262 VAP episodes were diagnosed in 209/936 patients (22.3%) who underwent mechanical ventilation. Median duration of mechanical ventilation was 3 days (interquartile range 2 to 6 days) among patients without VAP and 11 days (6 to 19 days) among patients with VAP. Late-onset VAP accounted for 61% of all episodes. The VAP rate was 37.6 episodes per 1,000 days at risk (95% confidence interval 33.2 to 42.4). The median daily nurse-to-patient ratio over the study period was 1.9 (interquartile range 1.8 to 2.2). By multivariate Cox regression analysis, we found that a high nurse-to-patient ratio was associated with a decreased risk for late-onset VAP (hazard ratio 0.42, 95% confidence interval 0.18 to 0.99), but there was no association with early-onset VAP.

**Conclusion:**

Lower nurse-to-patient ratio is associated with increased risk for late-onset VAP.

## Introduction

Ventilator-associated pneumonia (VAP) is the most frequent preventable adverse event affecting critically ill patients [1]. It occurs in approximately 25% of patients undergoing mechanical ventilation, for a rate of 4 to 25 episodes per 1,000 ventilator-days. Previous research has yielded conflicting results on attributable mortality, and reports range from 0% to as high as 70% [2-4]. VAP prolongs length of stay by up to 50 days, duration of mechanical ventilation by 5 to 7 days, and generates substantial extra costs, in the order of US$10,000 to 40,000 per episode [2,5,6].

Risk factors for VAP are still poorly understood and many have been described, including reintubation, duration of mechanical ventilation, intubation route, underlying pulmonary disease, use of H_2 _blocking agents, timing of tracheotomy, failed subglottic aspiration and low intracuff pressure [2,7,8]. Furthermore, the aetiopathogenesis of VAP has not been fully elucidated, and there is much debate and research into the origin of the micro-organisms that are involved in VAP and consequently into preventative measures [2,7-10].

At a time of universal cost containment policies, there is growing evidence that high workload or low staffing level increases the risk for negative patient outcomes [11,12], such as death [13] and nosocomial infection [12,14-16]. In a previous study [17] we investigated the association between nurse workload and infection risk in a medical intensive care unit (ICU) [17]. We estimated that a higher nurse-to-patient ratio was associated with a 30% risk reduction for all ICU-acquired infections, and that maintaining a nurse-to-patient ratio above 2.2 would ultimately lead to avoidance of a large proportion of all infections (population attributable fraction 26.7%).

The present work extends the former study by focusing on the main infection that occurs in the ICU, namely pneumonia, with the aim to determine whether workload influences the risk for VAP and whether this effect is similar for early-onset and late-onset VAP.

## Materials and methods

### Setting and study design

This prospective observational cohort study was conducted in the medical ICU of the University of Geneva Hospitals. The study design has been reported elsewhere [17]. In brief, all patients admitted from January 1999 to December 2002 were followed from admission to discharge. Collected variables included patient characteristics, admission diagnosis, Acute Physiology and Chronic Health Evaluation (APACHE) II score [18], length of stay, comorbidities and Charlson index [19], daily exposure to invasive devices, daily number of patients and nurses on duty, nurse training level, all-site nosocomial infections and daily individual PRN (Projet de Recherche en Nursing; a surrogate for the nursing acuity score) [20]. The protocol for preventing VAP remained unchanged throughout the study period.

### Definition of ventilator-associated pneumonia

Pneumonia was defined according to modified criteria proposed by the US Centers for Disease Control and Prevention [21-24]. This definition requires two of the following criteria to be satisfied: fever (increase of ≥ 1°C or body temperature > 38.3°C); leucocytosis (25% increase and a value ≥ 10,000 mm^3^), or leukopenia (25% decrease and a value ≤ 5,000 mm^3^); and purulent tracheal secretions (> 25 neutrophils per high-power field). It also requires one of the following to be satisfied: new and persistent infiltrates on chest radiograph; same micro-organism isolated from pleural fluid and tracheal secretions, or radiographic cavitation, or histological proof of pneumonia; or positive cultures from bronchoalveolar lavage (≥ 10^4 ^colony-forming units/ml). Pneumonia was considered to be VAP if it occurred from the day following intubation to five days after extubation. This period was deemed to be the time at risk. VAP was defined as early-onset when it occurred one to five days after intubation, and late-onset when it occurred from day six. Respiratory infections other than VAP were excluded from the analysis.

### Definition and measurement of nurse-to-patient ratio and other covariates

The way in which nurse-to-patient ratio was measured and consolidated is described in a previous report [17]. The ratio was determined by dividing the total number of nurses working during a given day by the patient census for that day. Assuming that the number of nurses per morning, evening and night shift was 13, 8 and 7, respectively, and the patient census was 15, the 24-hour nurse-to-patient ratio was 1.9, and the mean ratio per shift was 0.6 (1.9 divided by three shifts). We showed in the same study that a lower staffing level on a given day was associated with increased infection risk two to four days later. For this reason, we allowed for a latent period between exposure and outcome. Finally, because the precise time of contamination is unknown and incubation periods vary, the daily nurse-to-patient ratio for a given patient was consolidated as the mean of the ratios of the two to four preceding days. Other time-varying covariates (for instance, exposure to antibiotics) were consolidated in the same way with respect to timing. Consequently, exposures were allowed to change over time, and the two days preceding the infection or the end of the at-risk period were not considered.

### Statistical analysis

Infection rates were reported as the number of episodes per 1,000 days at-risk, with corresponding 95% confidence intervals (CIs), based on the Poisson distribution. Categorical variables were compared by χ^2 ^test; continuous variables were compared using a nonparametric test. The association between potential risk factors and infection was investigated using time-dependent Cox regression models and summarized by proportional hazard ratios (HRs) [25]. The main risk factor was nurse-to-patient ratio, consolidated as described above [17]. Patients without VAP were censored at the end of the at-risk period. Only the first episode of VAP was considered in a single failure per subject analysis, and days after this first episode were excluded from the time at-risk. The first analysis included all first episodes of VAP; the second analysis included only early-onset VAP; and the last analysis included only late-onset VAP. When failure was early-onset VAP, patients with a late-onset VAP were excluded and *vice versa*. Early-late and late-onset VAP were investigated in a univariate and multivariate model, and only variables associated with a *P *< 0.2 in univariate analysis were explored in multivariate analysis; only those with a *P *< 0.05 were considered statistically significant and retained in the final model. We looked for a threshold effect by categorizing the nurse-to-patient ratio in four groups, the cutoff values being arbitrarily the 25th, 50th and 75th percentiles of the ratio's distribution, and compared models using the likelihood ratio test. Analyses were conducted with STATA software (version 9.0; STATA Corp, College Station, TX, USA).

## Results

Of 2,470 patients followed during their ICU stay, 262 episodes of VAP were diagnosed in 209 out of 936 patients (22.3%) who underwent mechanical ventilation. A total of 172 patients experienced one episode of VAP and 37 patients experienced more than one episode. Late-onset infection accounted for two-thirds of VAP (160/262 [61%]). The VAP rate was 37.6 episodes per 1,000 days at risk (95% CI 33.2 to 42.4). The main characteristics of the study population are presented in Table 1. Compared with patients who did not suffer VAP, those with VAP stayed significantly longer in the ICU and required mechanical ventilatory support for a longer period. ICU mortality rates among patients with and those without VAP were 33.5% and 31.2%, respectively (*P *= 0.54), whereas hospital mortality rates were 45.0% and 39.5% (*P *= 0.154).

**Table 1:** Characteristics of the study population

Characteristic	Total population	Patients with VAP	Patients without VAP
Number of patients	936	209	727
Age (years)	65 (50 to 75)	64 (54 to 74)	65 (48 to 75)
Male sex	552 (59.0)	125 (59.8)	427 (58.7)
Charlson score at admission^a^			
0	335 (35.8)	69 (33.0)	266 (36.6)
1 to 2	326 (34.8)	91 (43.5)	235 (32.3)
3 to 5	184 (19.7)	34 (16.3)	150 (20.6)
> 5	91 (9.7)	15 (7.2)	76 (10.5)
APACHE II score at admission^b^			
< 26	367 (40.3)	74 (37.2)	293 (41.2)
26 to 30	170 (18.7)	37 (18.6)	133 (18.7)
> 30	373 (41.0)	88 (44.2)	285 (40.1)
Nursing acuity score at admission	226 (199 to 226)	226 (200 to 226)	226 (196 to 226)
Admission diagnosis			
Infectious disease^a^	238 (25.4)	70 (33.5)	168 (23.1)
Cardiovascular condition	315 (33.7)	76 (36.4)	239 (32.9)
Pulmonary disease	139 (14.9)	27 (12.9)	112 (15.4)
Other^a^	244 (26.1)	36 (17.2)	208 (28.6)
ICU stay (days)^a^	6 (3 to 11)	15 (10 to 23)	5 (3 to 8)
Duration of mechanical ventilation (days)^a^	3 (2 to 8)	11 (6 to 19)	3 (2 to 6)
ICU mortality	297 (31.7)	70 (33.5)	227 (31.2)
Hospital stay (days)^a^	18 (7 to 38)	29 (17 to 67)	14 (5 to 32)
Hospital mortality	381 (40.7)	94 (45.0)	287 (39.5)

Values are expressed as numbers (%) or median (interquartile range) for continuous variables. ^a^Significant difference between patients with and without VAP (*P *< 0.05). ^b^Twenty-six missing values. APACHE, Acute Physiology and Chronic Health Evaluation; ICU, intensive care unit; VAP, ventilator-associated pneumonia.

Microbiological documentation of the infection was obtained for 177 episodes (68%) in which 271 micro-organisms were identified; 74 infections (28.2%) were polymicrobial. The leading pathogens are summarized in Table 2.

**Table 2:** Distribution of leading pathogens in patients with ventilator-associated pneumonia

Details regarding episodes of infection	Total (%)^a^
Number of episodes microbiologically documented	177
Number of microorganisms identified	271
Gram-negative microorganisms	140 (52)
*Pseudomonas aeruginosa*	25 (9)
*Enterobacter *spp.	20 (7)
*Escherichia coli*	17 (6)
*Klebsiella pneumoniae*	15 (6)
*Proteus mirabilis*	7 (3)
*Haemophilus influenzae*	6 (2)
Non-aeruginosa *Pseudomonas *spp.	6 (2)
*Serratia marcescens*	5 (2)
Other Gram-negative micro-organisms^b^	39 (14)
Gram-positive microorganisms	75 (28)
*Staphylococcus aureus*	55 (20)
Coagulase-negative staphylococci	10 (4)
*Enterococus faecalis*	3 (1)
*Streptococcus pneumoniae*	2 (1)
Other streptococci	2 (1)
Other Gram-positive micro-organisms	3 (1)
Other micro-organisms	56 (21)
*Candida albicans*	34 (13)
Non-albicans *Candida *spp.	6 (2)
Other micro-organisms^c^	16 (6)

^a^The percentage given is that of the total number of micro-organisms (*n *= 271). ^b^Other Gram-negative microorgamisms included other enterobacteriaceae, *Acinetobacter *spp., *Citrobacter *spp., other *Klebsiella *spp. and *Morganella morgani*. ^c^Other micro-organisms include fungi and viruses.

The median daily nurse-to-patient ratio over the study period was 1.9, and ranged from 1.4 to 5.3 (interquartile range [IQR] 1.8 to 2.2). The median (IQR) ratios during the morning, evening and night shifts were 0.8 (0.7 to 0.9), 0.6 (0.5 to 0.7) and 0.6 (0.5 to 0.6), respectively. The median (IQR) nurse-to-patient ratio for a given patient was 2.0 (1.9 to 2.1) and the median (IQR) minimum and maximum values were 1.7 (1.6 to 1.8) and 2.3 (2.1 to 2.6), respectively. The crude HR (95% CI) of nurse-to-patient ratio 2 to 4 days before VAP onset was 0.64 (0.39 to 1.06) for all VAP episodes, 0.77 (0.42 to 1.40) for early-onset VAP episodes, and 0.43 (0.18 to 1.02) for late-onset VAP episodes. Results were similar in multivariate analysis for all VAP episodes (adjusted HR 0.66, 95% CI 0.40 to 1.10) and early-onset VAP episodes (adjusted HR 0.78, 95% CI 0.42 to 1.45). In multivariate analysis, higher nurse-to-patient ratio was associated with a reduced risk for late-onset VAP (adjusted HR 0.42, 95% CI 0.18 to 0.99). We identified no interaction between staffing level and nurses' training level. Neither the nurse training level nor the APACHE II score at admission had an effect on the hazard of VAP; the nursing acuity score at admission increased infection risk (Table 3).

**Table 3:** Risk factors for ventilator-associated pneumonia: crude and adjusted effect of staffing level

Risk factor	Early-onset VAP		Late-onset VAP	
	
	Crude HR (95% CI)	Adjusted HR (95% CI)	Crude HR (95% CI)	Adjusted HR (95% CI)
Nurse-to-patient ratio	0.77 (0.42 to 1.40)	0.78 (0.42 to 1.45)	0.43 (0.18 to 1.02)	0.42 (0.18 to 0.99)
Patient age	1.00 (0.99 to 1.01)	-	1.00 (0.99 to 1.01)	-
Male gender	0.92 (0.62 to 1.38)	-	1.15 (0.79 to 1.69)	-
Nursing acuity severity score	0.96 (0.92 to 1.01)	-	1.03 (1.00 to 1.07)	1.04 (1.00 to 1.08)
Charlson score	0.89 (0.81 to 0.99)	0.89 (0.80 to 0.98)	0.96 (0.87 to 1.05)	
Admission diagnosis				
Infectious disease	0.75 (0.46 to 1.20)	-	1.09 (0.75 to 1.60)	-
Cardiovascular condition	1.63 (1.10 to 2.41)	-	1.47 (0.97 to 2.23)	-
Pulmonary disease	0.77 (0.44 to 1.33)	-	0.56 (0.31 to 1.02)	0.53 (0.29 to 0.97)
Other	0.87 (0.53 to 1.41)	-	0.84 (0.49 to 1.43)	-
APACHE II score				
< 26	1	-	1	-
26 to 30	1.12 (0.67 to 1.86)	-	0.92 (0.49 to 1.72)	-
> 30	0.83 (0.53 to 1.31)	-	1.08 (0.70 to 1.67)	-
Nursing training level^a^	0.84 (0.60 to 1.18)	-	1.19 (0.91 to 1.56)	-
Invasive devices				
Central vascular line	1.50 (0.92 to 2.45)	1.71 (1.05 to 2.81)	3.06 (0.97 to 9.70)	4.14 (1.26 to 13.55)
Peripheral venous line	2.06 (0.76 to 5.61)	-	1.47 (0.95 to 2.29)	1.65 (1.06 to 2.59)
Peripheral arterial line	0.58 (0.32 to 1.06)	-	5.65 (0.78 to 40.84)	-
Urinary catheter	1.86 (0.66 to 5.26)	-	1.31 (0.41 to 4.16)	-
Nasogastric tube	1.40 (0.75 to 2.63)	-	2.39 (0.76 to 7.54)	-
Medication				
Parenteral nutrition	0.83 (0.49 to 1.41)	-	0.99 (0.65 to 1.51)	-
Therapeutic antibiotic	0.48 (0.32 to 0.73)	0.47 (0.31 to 0.71)	0.51 (0.29 to 0.91)	0.34 (0.19 to 0.62)
Prophylactic antibiotic	0.58 (0.18 to 1.84)	-	1.22 (0.56 to 2.63)	-
Gastric antacid drug	1.34 (0.90 to 2.00)	-	0.98 (0.66 to 1.46)	-

^a^Nursing training level is the number of intensive care unit certified nurses divided by the number of trainee nurses in critical care. APACHE, Acute Physiology and Chronic Health Evaluation; CI, confidence intervals; HR, hazard ratio; VAP, ventilator-associated pneumonia.

We then investigated a threshold effect of staffing level on the risk for late-onset VAP. We used the same adjustment variables as shown in Table 3 and categorized the nurse-to-patient ratio into four groups (≤ 1.8, 1.8 to ≤ 1.9, 1.9 to 2.2, and > 2.2), using the first group as baseline. The adjusted HRs (95% CIs) were 0.70 (0.41 to 1.18), 0.59 (0.36 to 0.95) and 0.54 (0.28 to 1.02), respectively, indicating a dose-response trend but no clear threshold. The fit of both models (using the staffing level as a continuous or a categorical variable) were similar (likelihood ratio test; *P *= 0.62).

## Discussion

This study confirms the high frequency of VAP in critical care and its negative impact on patient outcomes and resource utilization [5,6]. More importantly, our data contribute to a better understanding of the determinants of VAP. We demonstrated that a lower nurse staffing level increases the risk for late-onset VAP, independent of confounding factors (such as length of ICU stay or APACHE II score at admission), but it does not influence the occurrence of early-onset VAP.

We hypothesize that increased workload results in noncompliance with basic hygiene measures and infection control recommendations. During the past two decades, the number of nurses has decreased almost worldwide, whereas the level of patient acuity has increased [4,16]. Time constraints can increase the probability of error by creating a busy, stressful environment with distractions and interruptions [26], leading to low compliance with hand hygiene recommendations [27] and isolation procedures, or inadequate care for the ventilated patient. Cross-transmission of micro-organisms from one patient or the environment to another patient, or from one body site to another in the same patient, leads to colonization and infection. Because a large proportion of early-onset pneumonia results from early aspiration, it was not expected that staffing level would influence its occurrence. The observation that lower staffing level increases the risk for late-onset VAP is consistent with the multiple opportunities for cross-transmission during the course of patient care [28].

Although the need to specify critical nurse-to-patient ratios has grown in importance in health care research [29], there is no clear-cut staffing level threshold above which the infection risk decreases because the relationship between nurse-to-patient ratio and infection risk seems rather linear, as indicated in the present study and another one that was recently reported [17]. Indeed, there cannot be a single and unique threshold because the optimal staffing level depends on both risk and costs. Although the number of studies investigating the association between staffing level and preventable adverse outcomes is growing rapidly, few show how many or what proportion of infections could be prevented if the staffing level were modified, and to the best of our knowledge only three specifically examined healthcare-associated pneumonia. Two studies conducted in surgical ICUs [30,31] identified a significant increase in VAP and reintubation rates and costs if the nurse-to-patient ratio was below 0.5. Outside the ICU setting, an increase by one hour worked by registered nurses was associated with an 8.9% decrease in nosocomial pneumonia [32]. We recently reported that more than 20% of all-site ICU-acquired infections could be prevented, provided that the nurse-to-patient ratio was maintained above 2.2 [17].

Our study provided other interesting results. First, for several reasons, our VAP rate is higher than is usually found in the literature. Our surveillance system is prospective, on-site and consequently sensitive [33,34]; our case definition does not rely only on invasive diagnostic techniques; and the first two days following insertion of the endotracheal tube were excluded from the denominator because the patient, strictly speaking, is not at risk during these days. We previously highlighted the critical importance of the denominator in correctly expressing VAP rates [35]. Unlike others [36,37], we found no association between infection risk and nurses' training level, probably because we do not have recourse to 'pool' or 'float' nurses in the ICU. Interestingly, exposure to a peripheral vascular line was associated with an increased risk for infection. This should be considered a surrogate marker of severity of disease; the most severely ill patients will remain on the ventilator for a longer time and will be more likely to be exposed to several intravascular devices, including peripheral lines. We have no clear explanation for why patients admitted with a pulmonary disease experienced a lower VAP risk; one possibility is that a large proportion of these patients were ventilated for a short time for diseases such as asthma.

Our study suffers from some limitations. First, it was conducted in a single medical ICU, thus limiting the generalizability of the results. Second, we did not perform genotyping of microbial isolates to assess further the level of cross-transmission. Third, details of some process indicators that might have an adverse influence because of a lower staffing level (for instance, head positioning) were not routinely recorded. Fourth, as for any study on this topic, the challenge of accurately diagnosing VAP remains [2,38]. In our study, this diagnosis relied on standard definitions that are used worldwide, but it was not systematically supported by invasive diagnostic procedures such as bronchoalveolar lavage. There is undoubtedly some level of misclassification of outcome, with some conditions mistakenly considered as VAP and some true VAP episodes that physicians failed to recognize. However, this misclassification is quite independent of the staffing level, therefore being a random misclassification that would bias the estimate toward the null. Consequently, we are confident about the validity of our results.

Finally, an important limitation of the present study is that the exposure (nurse-to-patient ratio) is of an ecological nature, because all patients in the unit at any given time were exposed to the same ratio. Of note, this limitation affects all studies dealing with this topic [4,12,14], and how this bias affects the result is impossible to predict. In addition, the number of nurses on duty is determined in advance and cannot be fine tuned according to continuously changing patient conditions. Therefore, the ratio is a surrogate marker of workload and does not necessarily capture exactly what happens at the individual patient level. For instance, a given severely ill patient may be cared for adequately despite nurse shortage, because other nurses may come and help. However, increased workload should not be considered solely as an individual risk factor, because working conditions have impacts at the group level. For instance, several studies have demonstrated relationships between understaffing, job dissatisfaction, intention to leave, burnout, absenteeism and several preventable adverse events, including nosocomial infections [4,39,40]. This suggests that patient outcomes depend on both group and individual characteristics; consequently, the nurse-to-patient ratio may not precisely capture what happens at the individual level, but it does so at the group level.

Curtailing nurse staffing levels can lead to suboptimal care, which can raise costs far above the expense of employing more nurses [41]. On the other hand, there certainly remains room for improvement, regardless of staffing level. Questions about optimal staffing level and cost effectiveness remain unavoidable, and minimal nurse-to-patient ratios are already being demanded. For example, the governor of California announced that, by law (Assembly Bill 394), hospitals must have at least one licensed nurse for every six patients in medical-surgical units, with strict enforcement from 1 January 2004; in January 2005, this was modified to a ratio of one to five [42]. California was the first and, to date, only US state to pass such legislation. However, further research is still needed before concrete and evidence-based recommendations can be upgraded in guidelines for prevention of VAP, in terms of the strength of the evidence regarding nurse understaffing (grade II) [9]. Until then, given the heterogeneity of the sparse data in the literature, the ideal nurse-to-patient ratio should be estimated locally.

## Conclusion

This study shows that a low nurse-to-patient ratio increases the risk for late-onset VAP and provides further insight into the pathogenesis of VAP. It also adds to the growing body of evidence demonstrating that adequate staffing is a key determinant and a prerequisite for adequate care and patient safety.

## Key messages

• VAP is the most frequent adverse event affecting critically ill patients.

• Low nurse staffing level increases the risk for late-onset VAP.

• Adequate staffing is a prerequisite for high-quality care and patient safety.

## Abbreviations

APACHE = Acute Physiology and Chronic Health Evaluation; CI = confidence interval; HR = hazard ratio; ICU = intensive care unit; IQR = interquartile range; VAP = ventilator-associated pneumonia.

## Competing interests

The authors declare that they have no competing interests.

## Authors' contributions

SH developed the study design, coordinated its implementation, performed the data analysis and interpretation of results, and drafted the manuscript. DP and IU contributed to the study design, data analysis and writing of the manuscript. All authors read and approved the final manuscript.
